# Seed-Derived Microbial Community of Wild *Cicer* Seedlings: Composition and Augmentation to Domesticated *Cicer*

**DOI:** 10.1128/spectrum.02785-21

**Published:** 2022-05-31

**Authors:** Maya Lalzar, Adi Zeevi, Omer Frenkel, Abraham Gamliel, Shahal Abbo, Lilach Iasur Kruh

**Affiliations:** a Faculty of Natural Sciences, University of Haifa, Haifa, Israel; b Department of Biotechnology Engineering, ORT Braude College of Engineering, Karmiel, Israel; c Plant Protection, Plant Pathology and Weed Research, Agricultural Research Organization, Volcani Center, Israel; d Agricultural Engineering, Growing, Production and Environmental Engineering, Agricultural Research Organization, Volcani Center, Israel; e The Levi Eshkol School of Agriculture, The Hebrew University of Jerusalem, Rehovot, Israel; USDA San Joaquin Valley Agricultural Sciences Center

**Keywords:** *Bacillus*, *Cicer* spp., seed-borne bacteria, wild and domesticated plants

## Abstract

Seed-borne bacteria are a unique group of microorganisms capable of maintaining stable populations within plant tissues and seeds. These bacteria may benefit their host from germination to maturation and are of great interest for basic and applied plant-microbe interaction studies. Furthermore, many such beneficial bacteria present in wild plant species are missing in their respective congeneric domesticated forms. The objectives of this study were to explore the bacterial communities within the seeds of wild *Cicer* species and to select beneficial bacteria which could be used to improve production of domesticated chickpea (*C. arietinum*). We analyzed the composition of seed-borne bacteria of chickpea (*Cicer* spp.), comparing wild and domesticated species from different geographic locations. Subsequently, we isolated the dominant and prevalent seed-borne bacteria from wild *Cicer judaicum* and assessed their ability to colonize and affect the growth of domesticated chickpea and other legume crops. The composition and structure of seed-borne bacteria, determined by amplicon sequencing of the 16S rRNA gene, differed between wild and domesticated chickpea and varied among geographic locations. The genus *Burkholderia* dominated samples from domesticated chickpea at all examined sites, while *Bacillus* or *Sphingomonas* dominated cultures isolated from wild *C. judaicum*, dependent on geographic location. A particular *Bacillus* strain, *Bacillus* sp. CJ, representing the most prevalent bacterium in wild *C. judaicum*, was further isolated. *Bacillus* sp. CJ, applied by seed coating, successfully inhabited domesticated chickpea plants and improved plant growth parameters. These results demonstrate the potential for reconstructing the microbiota of crop plants using the wild microbiota reservoir.

**IMPORTANCE** Chickpea (garbanzo bean, hummus, *Cicer arietinum*) representing the third legume crop produced globally. As is the case for many other domesticated crops, the adaptation and resistance of chickpea to biotic and abiotic stresses is inferior compared to that of their wild progenitors and relatives. Re-establishing desirable characteristics from wild to domesticated species may be achieved by reconstructing beneficial microbiota. In this study, we examined the seed-associated microbiota of both wild and domesticated chickpea and applied isolated beneficial bacteria originating from wild *Cicer judaicum* to domesticated chickpea by seed coating. This isolate, *Bacillus* sp. CJ, was successfully established in the crop and enhanced its growth, demonstrating effective and efficient manipulation of the chickpea microbiota as a potential model for future application in other crop plants.

## INTRODUCTION

The phytobiome is composed of various microorganisms inhabiting different plant tissues, from seed to flower, interacting and affecting the plant’s growth and development (1). The bacteriome of seeds (i.e., the bacterial community inhabiting the surface and inner tissue of dry and developing seeds, excluding seed-borne pathogens) can serve as a vertically transmitted core microbiome of plants. This concept was demonstrated by Kim et al. (2), who showed that inheritance of seed-endophytic bacteria is a major path of microbial transmission across generations in rice, as well as a reservoir for beneficial bacteria that tightly interact with their host plant. Indeed, it has been shown that seed-associated bacteria fulfill significant roles in seed germination and plant growth (3, 4). In addition, some seed-associated bacteria can promote the host plant’s yield and biomass under biotic and abiotic stresses (3, 5–7). Hence, it has been suggested that the role of seed-associated bacteria as beneficial symbionts of mature plants is more significant than originally assumed (6, 8).

There are two possible ways for bacteria to inhabit seeds and occupy the plant following germination and during development: (i) vertical transmission, which refers to the transfer of endophytes from the mother plant to the offspring, is migration between generations, using an internal route through the nonvascular tissue of the mother plant to the seed or through the mother plant’s stigma; and (ii) horizontal transmission, which refers to environmental bacteria that encounter the mature seed in the soil (5, 8). The most intriguing communities are the bacteria which inhabit the seeds and are able to occupy the seedling’s inner tissues when it germinates. These interactions are affected by host filtration, i.e., plant host species or genotype on the one hand (9), and the bacteria’s ability to adapt to the plant’s inner environment on the other hand (10).

It has been claimed that conventional, intensive agricultural practices affect the interaction of bacteria with their host plants (6) for two main reasons. First, modern agriculture depends on selective breeding for desirable traits in crops, including favorable plant morphology, growth rate, yield, and disease resistance. The directed evolution of domesticated plants may have unintentionally caused a reduction in plant phenotypes and mechanisms which facilitate microbial symbiosis (11). For example, some domesticated species of the legume subfamilies Cercidoideae and Papilionoideae have lost their ability to form root nodules for inhabitation by nitrogen-fixing bacteria (12). Second, conventional intensive agriculture practices (e.g., monoculture, irrigation, tillage, fertilization, and use of pesticides and/or herbicides) alter soil physicochemical properties and microbiota composition (13–17). Thus, the repertoire from which seed-borne bacteria are acquired is altered and may be deficient in key (core) species (2). Furthermore, long-term practices may drive evolutionary processes among populations of soil bacteria and hence affect their phyto-symbiotic beneficial traits (18). In fact, long-term nitrogen fertilization has resulted in the evolution of less-mutualistic rhizobia, providing lower benefit to the host (19).

In a scenario of loss-of-function due to genetic barriers, the restoration of host-symbiont interactions may be difficult (particularly so for genetic loss/drift in the plant host). In contrast, associations lost due to agrosystem-related barriers may be restored by augmentation. It has been suggested that the reservoir of seed-associated bacteria in the wild ancestors of crops or other wild plants could be applied by seed treatment to domesticated crop plants in order to reconstruct beneficial bacteria-plant interactions (6). However, the outcomes of interaction between a specific bacterium and potential host (i.e., the domesticated counterpart or other crops) can vary in both the success of colonization and the effect on the plant (20, 21).

This study aimed to measure the extent of divergence of seed-borne bacteria between wild and domesticated plant species and to resolve the two possible barrier types. For this purpose, members of the genus *Cicer* (Papilionoideae: Fabales) were chosen as a model system. Members of Papilionoideae, a highly diverse subfamily of Leguminosae (22, 23), are renowned for their key role in the coupled terrestrial nitrogen and carbon cycles and their immense agronomic importance (24). *Cicer judaicum*, a distant wild relative of chickpea, is the only species of tribe Cicereae that occurs naturally in Israel, mostly in the central mountain belt. In certain locations, domesticated chickpea is grown alongside those natural habitats, often in close proximity to wild *C. judaicum* populations (25, 26). Hence, these sympatric wild populations and chickpea crops are good candidates for exploring the bacterial communities of both species and the ability of the wild taxon to serve as a reservoir of bacteria for the reconstruction of beneficial interaction symbiosis.

The objectives of the current study were as follows: (i) to characterize the seed-derived bacteriome associated with seedlings of both wild and domesticated *Cicer*; (ii) to identify core host-bacteria associations particular to the wild species and attempt their reconstruction in the domesticated species; and (iii) to examine the effect of plant species relatedness on interactions with specific microbes and their potential outcomes.

## RESULTS

### Bacterial community composition of wild and domesticated *Cicer*.

**FIG 1:**
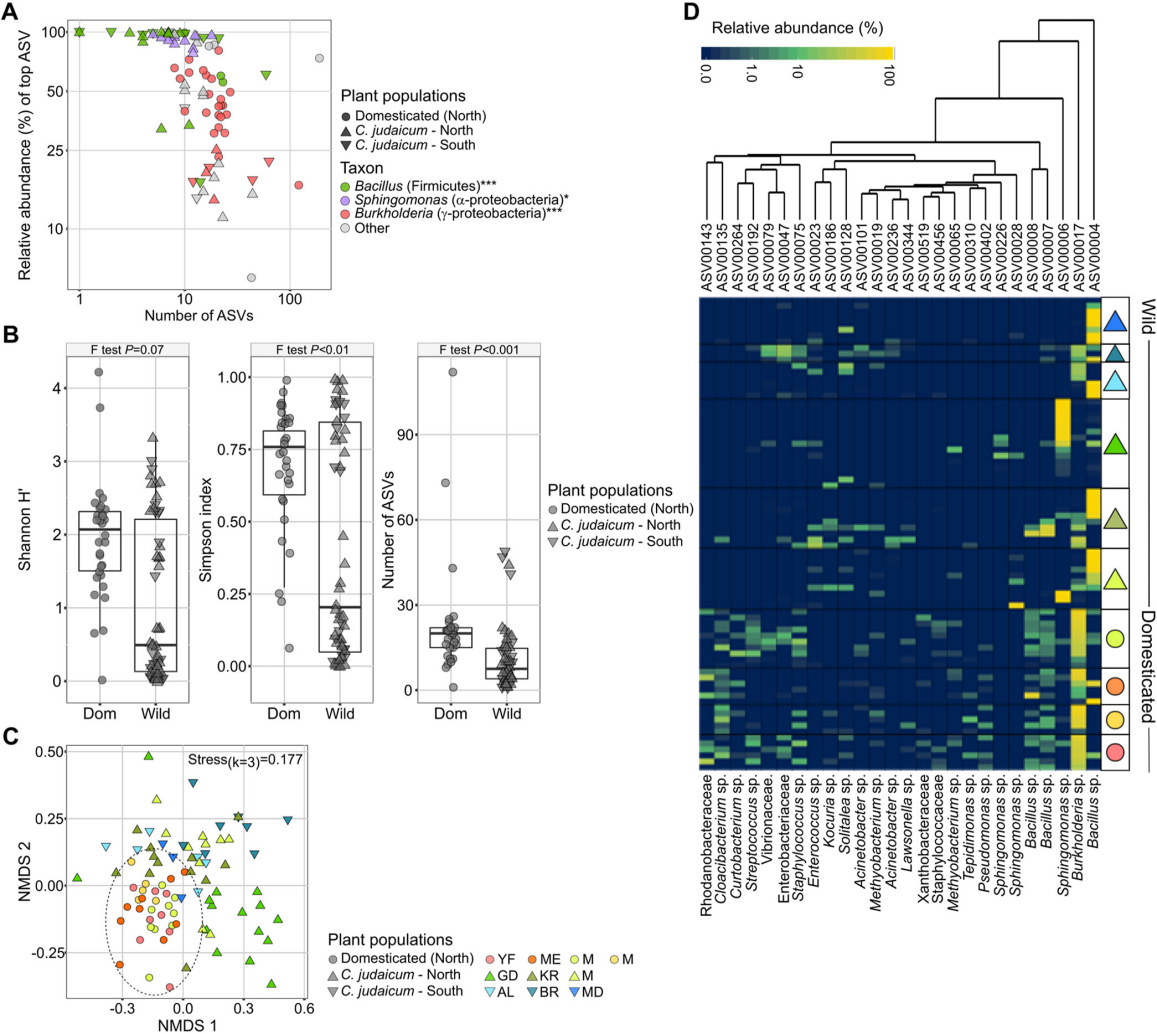
Bacterial community composition of wild and domesticated *Cicer* seedlings sampled in different regions across Israel. (A) Biplot representing relatedness between species richness (as number of amplicon sequence variants [ASVs]) and level of dominance (as relative abundance of the most dominant ASVs). ASVs belonging to three genera were frequently dominant. Asterisks represent significant enrichment of taxon dominance frequency in *C. judaicum* compared to that in domesticated *Cicer* (*Bacillus* and *Sphingomonas*), or in domesticated *Cicer* compared to that in *C. judaicum* (*Burkholderia*), based on the Fisher’s exact test. (B) Alpha diversity parameters (Shannon’s H’, Simpson index, and observed richness) were calculated based on rarified (*n *=* *450) ASV counts. Variances were compared using Levene’s test. (C) Non-metric multidimensional scaling (NMDS) analysis based on a Bray-Curtis dissimilarity matrix between samples. (D) Heatmap presenting the relative abundance of the 27 most prevalent ASVs. Taxonomic assignment of each ASV, up to genus level, is denoted below the heatmap. Shapes and colors on the right represent *Cicer* species and site, as in panel C.

In order to examine the composition of seed-borne bacteria able to establish in the germinated plants, seeds were germinated and grown under sterile conditions for 10 days. Based on high-throughput sequencing of the bacterial 16S rRNA gene, we found the seedlings’ bacterial communities to be characterized by low richness and high dominance. Indeed, in most cases, a single population (single amplicon sequence variant, ASV) accounted for >50% of the total bacteria. Figure 1A shows ASV richness versus ASV dominance. While the dominant ASVs in most of the wild *Cicer* seedlings belonged to *Bacillus* or *Sphingomonas*, the domesticated seedlings were dominated by *Burkholderia*. We used a Fisher’s exact test to compare the frequencies of seedlings dominated by each of these three groups between wild and domesticated *Cicer.* The test results showed a significantly higher frequency of *Bacillus* (*P < *0.0001) and *Sphingomonas* (*P < *0.05) in wild *Cicer* compared to that in domesticated *Cicer*, as well as a higher frequency of *Burkholderia* (*P < *0.0001) in domesticated *Cicer* compared to that in wild *Cicer*.

Most of the seedlings examined harbored >30 ASVs. However, even though the average values for dominance (represented by the Simpson index) and diversity (represented by the Shannon index) of wild and domesticated *Cicer* were similar, the values for wild *Cicer* were more broadly distributed (higher variance), resulting in two separate groups with either high or low dominance/diversity (Fig. 1B).

**FIG 2:**
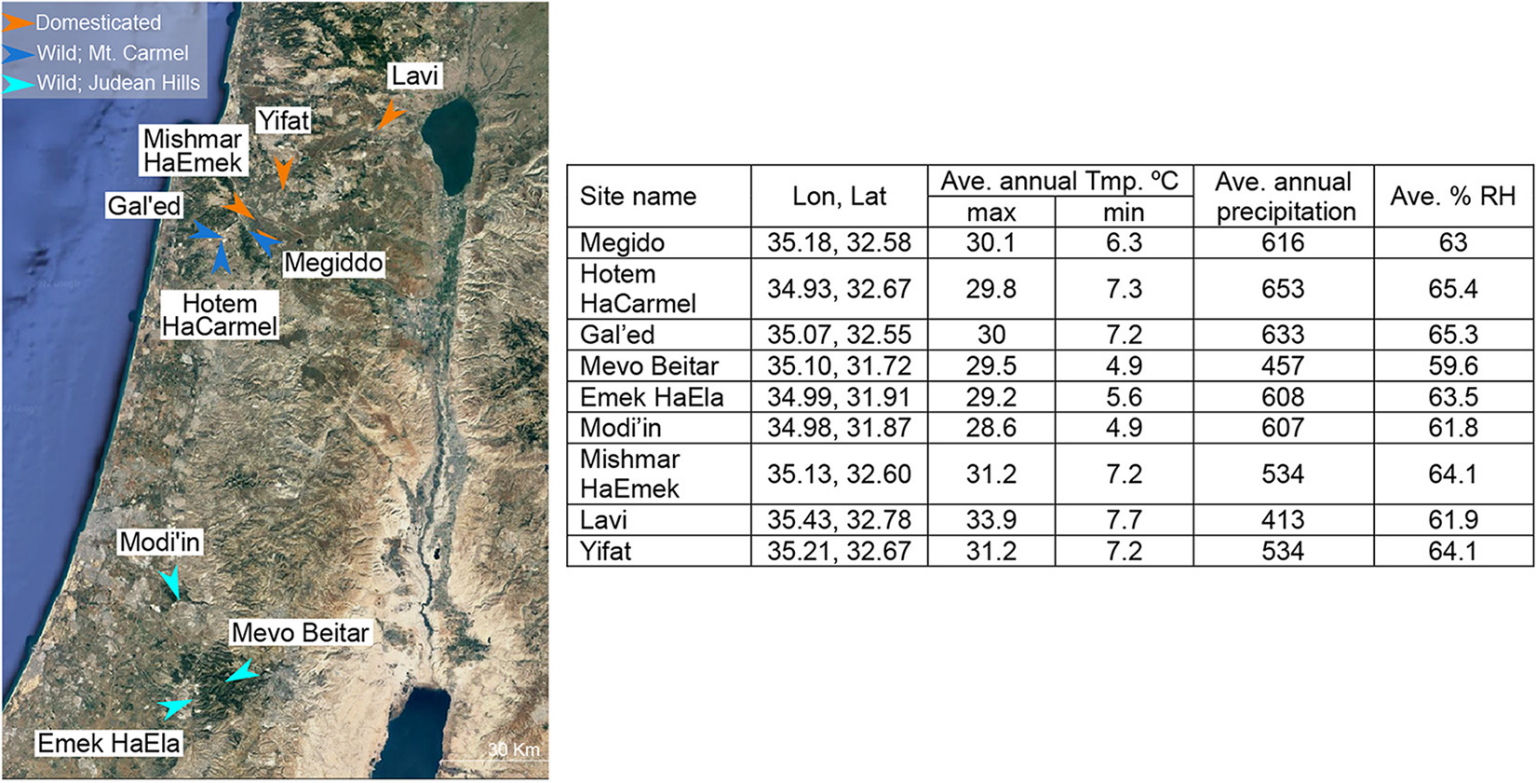
Map locations of the wild and domesticated *Cicer* seed samples.

Seed samples of domesticated *Cicer* were sampled across the Jezreel Valley in northern Israel, while wild *Cicer* seeds were collected along two transects (Mt. Carmel and the Judean Hills, Fig. 2). To compare compositions of seedling bacteriomes, we used a permutational analysis of variance (PERMANOVA). The results showed significant differences in seedling bacterial community compositions between domesticated *Cicer* and wild *Cicer* of either Mt. Carmel (*R* = 0.13, *P < *0.001) or Judean Hills (*R* = 0.19, *P < *0.001) origin. In addition, there were significant differences in the bacterial community composition of wild *Cicer* obtained from Mt Carmel compared to those from the Judean Hills (*R* = 0.05, *P < *0.01). Similar trends were demonstrated by non-metric multidimensional scaling analysis (NMDS) (Fig. 1C). A thorough examination of these populations (Fig. 1D) showed that a specific *Bacillus* strain, ASV00004 (Fig. 1D), was the most prevalent ASV (36/54) in wild *Cicer* and was the dominant ASV in 39% of the seedlings. This ASV was much less prevalent in domesticated *Cicer* (6/31) and was the dominant ASV in only 2 of 31 seedlings. *Sphingomonas* ASV00006 (Fig. 1D) was prevalent only in wild *Cicer* (wild 10/54, domesticated 0/31) and only at one specific sampling site (Gal’ed). In comparison, the most dominant and most prevalent bacterial population in domesticated *Cicer* belonged to *Burkholderia* ASV00017 (Fig. 1D) (24/31). In wild *Cicer*, *Burkholderia* was the dominant population in 7/54 seedlings, but its relative abundance never exceeded 25% (Fig. 1D). The modified stochasticity ratio (MST) was calculated based on Jaccard (wild MST = 0.28; domesticated MST = 0.43) and Bray-Curtis dissimilarity matrices (wild MST = 0.18; domesticated MST = 0.43). In both cases and for both species, MST values were <50%, indicating a dominance of deterministic processes in community assembly. However, MST values were significantly higher (Jaccard-based *P < *0.01; Bray-Curtis-based *P < *0.01) for the domesticated *Cicer* species compared to those of the wild one.

### Isolation of microorganisms from wild and domesticated *Cicer* seedlings.

Bacterial isolation was conducted from each seedling. Of the platings of seedlings originating from domesticated *Cicer*, 75% resulted in fungal growth on the nutrient agar (NA) plates, while wild *Cicer* plating resulted in bacterial growth only. Sixty morphologies were identified from all samples. One bacterial morphotype formed colonies on NA which had a round shape, irregular edges, and an opaque, creamy color. This was repeatedly isolated from various wild seedling specimens, and sequencing of the 16S rRNA gene of this isolate showed that it belonged to the Bacillus subtilis species, with a 100% match to the dominant strain found in bacterial NGS analysis of the wild *Cicer* (ASV00004) (Fig. 1D). We named this isolate “CJ.”

### Colonization of the CJ strain and its effect in different domesticated legumes.

**FIG 3:**
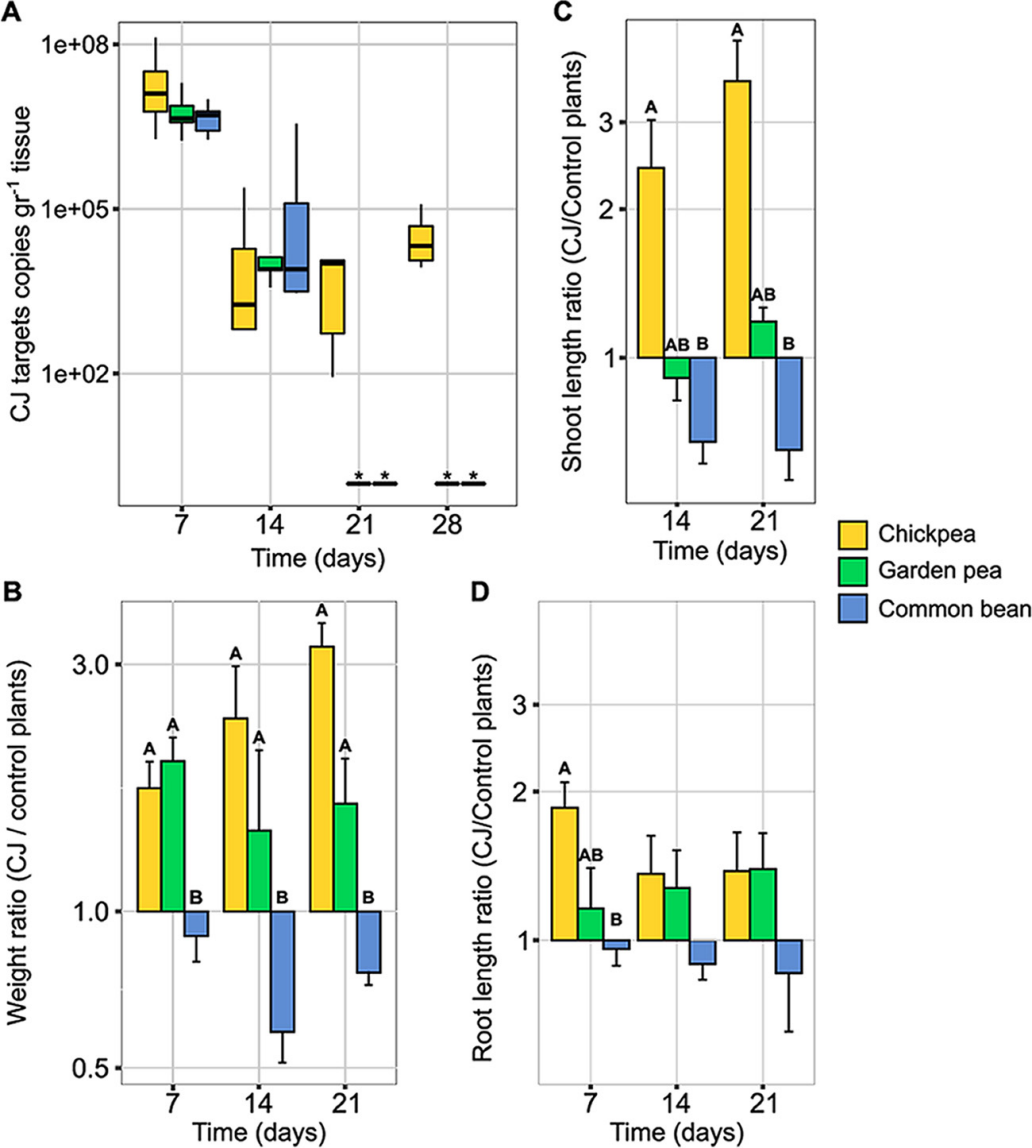
The ability of the CJ isolate from the wild *Cicer* seedling to inhabit plant tissue (A) and to affect plant weight (B), shoot length (C), and root length (D). Isolate CJ from wild *Cicer* was applied to domesticated legumes: chickpea (*C. arietinum*), garden pea (Pisum sativum), and common bean (Phaseolus vulgaris) seeds (*n *=* *15 per plant species). Control plants were germinated under the same conditions without bacterial coating (*n *=* *15 per plant species). Plants were sampled 7, 14, and 21 days post-sowing. Values represent mean and standard deviation of the ratios between treated and control plants. Different letters denote significant differences (Tukey’s honestly significant difference test, α = 0.05).

Because the CJ strain was naturally abundant among the wild *Cicer* samples and rarely detected in domesticated *Cicer*, as demonstrated by NGS analysis, its ability to inhabit and affect chickpea and other domesticated legumes was examined by seed coating. The isolate quantity in plant tissues grown from seeds coated with the CJ strain was examined by quantitative PCR (qPCR) specific primers. In addition, stem and root length and weight were measured. The isolate’s persistence in chickpea was longer than that in pea or common bean (28 versus 14 days, respectively; Fig. 3A). The isolate’s effect on plant biomass was also significantly more beneficial in chickpea compared to that in pea and was disadvantageous in common bean (Fig. 3B). A similar effect was observed for shoot length, but not for root length (Fig. 3C and D).

## DISCUSSION

Bacteria associated with seeds attract much attention, since their lifestyle makes them excellent candidates for seed coating: a well-established technique widely accepted by farmers and easy to apply in agroecosystems. Therefore, bacteria that originate from seeds and establish in seedlings and mature plants have high potential for biocontrol and/or biofertilization purposes (6, 8, 27). Indeed, Matsumoto et al. (7) demonstrated that seed-borne Sphingomonas melonis which inhabit disease-resistant rice and are vertically transmitted by inheritance confer resistance to disease-susceptible rice cultivars.

This study examines the microbiota of germinated seeds of wild and domesticated *Cicer* species collected from various environments in Israel (Fig. 2). The composition and structures of seed-borne bacteria differed between the two *Cicer* species and varied among geographic locations (Fig. 1). Our results are similar to those of Özkurt et al. (28), who examined seedlings colonized by seed-associated microbes. They showed different bacterial community composition and structure between wild emmer wheat (*Triticum dicoccoides*) and domesticated bread wheat (Triticum aestivum). In both studies, because seeds were collected from different locations and directly germinated, the species and environmental effects (e.g., climatic, geographic, edaphic, agrosystem practice-related) are confounded. Nevertheless, both studies demonstrate a common feature of seed-derived seedling bacterial assemblages: high seed-to-seed variation in composition. Similarly, Rosenblueth et al. (29) reported high seed-to-seed variability in microbiota composition, even within the same cob (maize) or pod (Phaseolus vulgaris). In rice seeds, by contrast, low variability was observed across 99 different subspecies (30). This raises the question of the mechanisms governing the assembly of seed microbiota and the balance between deterministic (e.g., selection) and stochastic (e.g., ecological drift) processes (31, 32). In our study, as in the study by Guo et al. (30), the calculated estimates of stochasticity (MST) indicated a divergence toward deterministic processes in both *Cicer* species, but significantly lower stochasticity in the wild species than in the domesticated one. This is exemplified by the more pronounced effect of geographic location in the wild species compared to that in the domesticated species (Fig. 1C).

One aspect distinguishing wild from domesticated samples was community structure: diversity values had wider distribution among wild *Cicer* samples relative to that among domesticated ones (Fig. 1B). In fact, in specimens of wild *Cicer* occupied by either *Bacillus* spp. or *Sphingomonas* spp., dominance was high and diversity was low. Otherwise, the dominance was lower and the diversity much higher. This phenomenon was not characteristic of domesticated *Cicer*. Comparisons between community structures of different species of seeds have been reported (28, 33); however, to the best of our knowledge, such a divergence in distribution patterns has not been reported.

Another aspect distinguishing wild from domesticated species was community composition: *Bacillus* or *Sphingomonas* dominated wild *Cicer* seedlings, dependent on geographic origin, while *Burkholderia* dominated the domesticated *Cicer* at all sites examined. Several studies have shown that the bacterial community composition in seeds is affected by environmental factors (5, 28, 34, 35) and plant host species, including domestication processes (33, 36–38). Nevertheless, in many of these cases, these factors were confounded. Since the pod maturity season is different between the *Cicer* species (wild *Cicer* were collected in the spring while domesticated chickpea pods were sampled in summertime), seasonality may also have been a factor in the differentiation of bacterial community composition, as was demonstrated in the microbiota of *Brassica napus* seeds (39). Importantly, the observed differences between these two *Cicer* species suggest that key bacterial associates of wild *Cicer* are missing or rare in the domesticated *Cicer*, offering suitable candidates for augmentation in domesticated plants: the most dominant and most prevalent seed-borne bacterium of wild *Cicer*, *Bacillus subtills* CJ strain, was isolated. Its prevalence in domesticated *Cicer* was 6.5 times lower than that in wild *Cicer*, and it was rarely dominant when present there (Fig. 1D). Using seed-coating techniques, *B. subtills* CJ was successfully established in the stems and roots of domesticated chickpea plants as an endophyte (Fig. 3). Hence, the genetic differences between the two *Cicer* species did not pose a barrier for colonization and establishment of isolate CJ. These results support the claims of Berg and Raaijmakers (6) and Wassermann et al. (34) that reservoirs of seed-associated bacteria from the wild relatives of crops can be applied by seed treatments in order to reconstruct bacteria-plant interactions in the crop species. Furthermore, we demonstrated increases in biomass and the root and shoot length of domesticated chickpeas, fulfilling the purpose of beneficial interaction. Similarly, endophytic bacteria from the wild shrub *Dodonaea viscosa* were able to enhance the growth of canola (*Brassica napus*) plants by seed inoculation (40).

In our study, phylogenetic proximity of wild and domesticated plants seemed to be essential for successful application (Fig. 3). To examine broader applicability, we need to consider bacterium-host interactions and their specificity. Plant-bacteria interactions may be generalist (41, 42) or specific in nature (43). After occupying the plant, a specific bacterium strain may produce beneficial relationships with a certain plant host while damaging other plant taxa (20, 21). In this study, *B. subtills* CJ’s ability to occupy the plant varied among legume species. Furthermore, the effect of this isolate on the plant biomass was significantly more beneficial in chickpea than in pea, and was disadvantageous to common bean. The molecular networks and genetic underpinnings of successful plant-bacteria interactions have been elucidated only for a few cases. In legumes, interactions with nitrogen-fixing endophytic rhizobia are well studied and demonstrate the coevolution of plant and symbiont (44). Vargas et al. (45) showed a species-specific interaction between two rice cultivars and two beneficial diazotrophic bacterial strains, demonstrating different patterns of expression of plant ethylene receptors, dependent on both plant and bacterial genotypes. On the other hand, a set of 24 general non-self-response genes were identified in *Arabidopsis* in response to a diverse array of bacterial inoculants (46). This core set included genes which are involved in tryptophan-derived secondary metabolism production, cell wall-associated proteins, and plant receptor genes connected to signal incorporation. A recent paper described the mechanism of disease suppression in rice by a vertically inherited symbiotic Sphingomonas melonis (7). This bacterium produces anthranilic acid *in planta*, resulting in the suppression of the seed-borne pathogen Burkholderia plantarii. Also recently, for *Bacillus subtillis* NCIB3610 applied to melon seeds, the mechanism of plant growth promotion was connected to the secretion of an extracellular matrix containing amyloid protein and fengycin (47). In accordance, a future examination of reciprocal *B. subtillis* CJ-legume responses may shed light on the mechanisms underlying the differences in colonization and effects among the phylogenetically related plant hosts examined here.

Interestingly, the order of *B. subtillis* CJ-legume interaction outcomes was related to phylogenetic distances among plant species: ([wild C. judaicum: C. arietinum]: Pisum sativum): Phaseolus vulgaris (48). Furthermore, garden pea, like chickpea, is a cool season legume domesticated from a Near Eastern annual. In eastern Turkey, the progenitor of domesticated pea often grows side by side with wild chickpea species (49, 50), similar to the occurrence of wild *Pisum* and wild *Cicer* in many locations in Israel (51). Likewise, domesticated pea and domesticated chickpea are grown in rotation in many world regions. The common bean is a warm-season crop domesticated in Mesoamerica, but in recent centuries it has become part of the crop repertoire in the Mediterranean basin. Therefore, the null hypothesis would be that garden pea will resemble chickpea, while common bean will behave differently, as was indeed demonstrated in terms of bacterium-host interactions.

Kim et al. (2) demonstrated weak but significant phylosymbiosis relations (i.e., closely phylogenetic host species that harbor microbiota of similar composition [52]) between seed-borne bacterial endophytes and their host, rice. The number of legume species used in our study is too small to assess and measure relatedness between host-symbiont interactions and phylogenetic distance. Such future information could be highly valuable for applicative and basic knowledge purposes and should be further examined.

## MATERIALS AND METHODS

### Plant material.

Wild Cicer judaicum seeds (here, wild *Cicer*) were sampled from several locations during April 2018. Seeds from domesticated chickpea (Cicer arietinum; here, domesticated *Cicer*) were sampled from commercial fields during July 2018 (see details in Fig. 2). Following transportation to the laboratory, all seeds were stored in paper bags and dried in an incubator at 27°C for 10 days within the pods. Next, dried seeds were stored at 4°C for an additional 10 days (53).

### Seed germination under sterile conditions.

Seeds were germinated under sterile conditions on a water-agar medium. Bacteriological agar (Neogen, Lancashire, United Kingdom) 0.8% in tap water was sterilized by autoclave and poured into a sterile 50-mL tube (25 mL per tube). After cooling to room temperature, seeds were placed on top of the agar surface using sterile tweezers (one seed per tube), and the caps were sealed and placed at 28°C. For wild *Cicer* seeds, the seed coat was scarified using sterilized tweezers prior to placement, and these seeds were allowed to germinate for 10 days. During incubation, the agar was examined for signs of contamination by bacteria and/or fungi and contaminated tubes were immediately discarded.

After this, the 10-day-old seedlings were removed from the tubes. Each seedling was separately homogenized in 900 μL of sterilized double-distilled water using a sterilized mortar and pestle. Homogenates were then placed in a sterile 2-mL Eppendorf tube.

### Isolation of seed-borne bacteria inhabiting seedlings.

We were interested in bacteria which originate from seeds and can effectively colonize the developing plant. Therefore, we examined the bacterial communities which developed in the seedlings germinated under aseptic conditions.

For isolation of bacteria, 500 mL of seedling homogenates was serially diluted in sterile saline up to 10^6^ dilution. Aliquots of 0.1 mL were spread on nutrient agar (NA) petri plates (Neogen, Lancashire, United Kingdom) containing peptone 5.0 g/L, meat extract 3.0 g/L, sodium chloride 8.0 g/L and agar 12.0 g/L and incubated for 48 h at 27°C. This medium was chosen because the dominant bacteria which were identified as being associated with *Cicer* seedlings by NGS were found to be chemoheterotrophic. Because in this study the pods were opened directly into a sterile germination system, it was assumed that all the isolated bacteria had originated directly from the seed.

After incubation, bacterial colonies were counted, and the presence of fungi was recorded. The different colonies were characterized by their morphology and each morphology was subjected to a separate NA plate to produce pure culture. Each pure culture was grown on nutrient broth (NB) and, after its purity had been confirmed by isolation plating, was stored at −80°C in 50% glycerol stock.

Identification of the isolates obtained from wild *Cicer* (CJ strain) was conducted by sequencing the 16S rRNA gene: a single colony of each isolate was collected using a sterile loop and subjected to a PCR mix containing 12 μL double-distilled water (DDW), 10 μL PCR mix (Kodaq 2× PCR Master Mix with dye, Applied Biological Materials, Richmond, Canada) and 1 μL of each primer (10 pmol) targeting the 16S rRNA gene (27F, AGAGTTTGATCMTGGCTCAG; 1513R, ACGGYTACCTTGTTACGACTT). The PCR procedure was as follows: DNA was denatured at 95°C for 5 min, followed by 30 cycles each of 95°C for 30 s, 58°C for 30 s, and 72°C for 1 min, followed by 5 min at 72°C (54). The PCR product was sequenced by the Sanger method at Hy Laboratories (Rehovot, Israel).

### DNA extraction from plants and 16S rRNA amplicon sequencing.

Plant tissues (stem and root/seedling) were weighed and homogenized with lysis buffer (Plant/Fungi DNA Isolation kit, Norogen Biotek Corp., Thorold, Canada) in a 1:2 ratio using a sterilized mortar and pestle. DNA extraction was accomplished using a Plant/Fungi DNA Isolation kit following the manufacturer’s instructions. DNA quantity and quality were examined using a NanoDrop spectrophotometer (NanoDrop 2000/2000c; Thermo Fisher Scientific, Waltham, MA). The extracted DNA was used for amplicon sequencing and qPCR. In order to describe the microbiota of the germinated seeds and examine their composition, we amplified and analyzed partial 16S rRNA gene sequences. Genomic DNA was used a template for the amplification of a 292-bp fragment spanning the V4 variable region of the small subunit rRNA gene. PCR was performed using the primers 515F and 806R, as previously described (55). A two-stage targeted amplicon sequencing protocol was used, as previously described (56). Libraries were loaded onto a MiniSeq flow cell and sequenced (2 × 150 paired-end reads) using an Illumina MiniSeq sequencer. PCR amplifications library preparation, and sequencing were performed at the University of Illinois at the Chicago Sequencing Core.

### Sequence data processing and analysis.

Sequence data were analyzed using the DADA2 pipeline (57). Fastq-formatted reads were trimmed and filtered for low quality using the command ‘filterAndTrim’ with parameters maxEE = 2, maxN = 0, and trimleft = 20 constant and the trunclen parameter varying based on specific run-quality parameters. Error rate estimation was carried out using the ‘learnerror’ command with default parameters, but the randomize parameter was set to TRUE. Following this, the DADA2 algorithm was implemented for error correction, and a count table containing the amplicon sequence variants and counts per sample was produced. After merging the sequence data from all runs, suspected chimeras were detected and removed using the command ‘removeBimeraDenovo’ with the default parameters. For each ASV, taxonomy was inferred by alignment to the Silva nonredundant small subunit rRNA database (version 138) using the command ‘assignTaxonomy’ with the default parameters but with minimum bootstrap confidence value set to 80%. The ASV count table was then inspected and filtered: ASVs with sequences shorter than 250 bp or longer than 252 bp were removed. In addition, all ASVs of nonbacterial origin (including unclassified, Eukaryota, Archaea, chloroplast, and mitochondria) were removed.

Since the plant chloroplast and mitochondrial small subunit rRNA genes were successfully amplified using general bacterial primer pairs in PCR, reads originating from the plant commonly dominated the sequence pool in our samples. The bacterial reads per sample ranged between 93 and 66,118, with an average of 8,047 sequences. Therefore, in many cases, the bacterial read counts of specific samples were low. Hence, we employed the rarefaction approach in order to determine a minimal number of bacterial reads per sample to pass on to analysis. Indeed, for most samples, a plateau in the accumulation of ASV numbers was reached at very low read counts. We therefore used a cutoff of 450 bacterial reads per sample as the lower boundary for inclusion in further analyses. In total, 85 samples were retained, 54 from wild *Cicer* and 31 from domesticated *Cicer.* Altogether, 922 bacterial ASVs were included in the analysis (data not shown); prevalence and relative abundance in wild and domesticated *Cicer* were calculated for all ASVs. For each sample, the dominant ASV was denoted. Then, a Fisher’s exact test was conducted to compare the frequencies at which specific taxa dominated the bacterial communities of seedlings from wild and domesticated *Cicer*. Frequencies were considered significantly different when *P* values were <0.05. Fisher’s exact test was calculated using the ‘fisher.test’ function in the R package ‘stats.’ Alpha diversity parameters were calculated using the R ‘vegan’ package (version 2.5.7). The Shannon diversity index (H’), the Simpson index of evenness, and the number of ASVs were calculated based on a count table rarified to 450 sequences per sample. We compared variances between wild and domesticated *Cicer* for each of the three parameters using Levene’s test. Variances were considered significantly different when *P* values were <0.05. In order to assess differences in composition related to plant species and eco-geographic habitat, we used the permutational analysis of variance test implemented in the R package ‘vegan’. *Post hoc* pairwise comparisons were conducted using the pairwise PERMANOVA module in the R ‘pairwiseAdonis’ package. We further examined similarities in bacterial community composition using NMDS ordination. NMDS was calculated using the R package ‘vegan’ metaMDS command with Bray-Curtis distances, K = 3, and 1,000 tries. To assess the relative importance of stochastic and deterministic processes in bacterial community assembly, the modified stochasticity ratio was calculated. This metric reflects the modified ratio of the mean expected similarity in the null model to the observed similarity (31). MTS values below 50% denote a dominance of deterministic processes, and MST values above 50% denote a dominance of stochastic processes. MST values were calculated using the R package ‘NST’ (version 3.0.6) based on the Jaccard distance matrix among samples using presence-absence information and based on Bray-Curtis dissimilarities, including relative abundance weights. MST values were compared between wild and domesticated *Cicer* communities using the bootstrap method implemented in the ‘NST’ R package.

### Examination of colonization of domesticated legumes by isolate CJ.

The three plant species belonging to the superfamily Papilionoidea were chosen for a common-garden experiment: chickpea (*C. arietinum*), garden pea (Pisum sativum), and common bean (Phaseolus vulgaris). All three are domesticated representatives of their respective genera and tribes and are grown commercially in Israel. Furthermore, based on phylogenetic analysis, *C. arietinum* and *P. sativum* are more closely related to each other than they are to P. vulgaris (48).

Isolate CJ was grown on NA plates overnight at 28°C. Then, colonies were diluted with DDW until optical density at 600 nm reached 0.1 (approximately 1 × 10e^6^ CFU/mL). Since this bacterium originated from germinated seeds, a seed-coating technique was chosen as the bacterization method. Seeds of the three plant species were submerged in the bacterial culture for 1 h and then sowed in vermiculite medium for germination in a growing room with natural daylight. The plants were watered when needed, and 5 plants were sampled every 7 days. As a negative control, 5 plants originating from seeds that were not submerged with bacteria were examined at each time point. At sampling, the lengths of the roots and shoots of each plant were measured and the whole plant was weighed. Then, genomic DNA of each plant was extracted using the Plant/Fungi DNA Isolation kit (Norogen Biotek Corp.). The experiment was conducted 3 times in order to assess the repeatability of these results. The effect of augmentation of isolate CJ was estimated by calculating ratios between CJ-inoculated plants and the equivalent un-inoculated controls. For weight, differences in ratios were arcsine-transformed and compared among plants by analysis of variance (ANOVA) with a *post hoc* Tukey’s test. For root and shoot lengths, ratios were log-transformed, and Kruskal-Wallis tests followed by *post hoc* Dunn’s tests were conducted to compare the effect of CJ inoculation among plant species.

### CJ-specific primer design and qPCR analysis.

Since *Bacilli* species naturally inhabit legumes and their colony morphology is common, there was a concern that live counts of the bacteria associated with the plant would not be accurate enough. Therefore, designing a specific quantitative assay was necessary. For this purpose, genomic DNA was extracted from pure isolate CJ culture and sequenced on an Illumina MiSeq platform (pair-ends, 2 × 150 bases). A draft genome was assembled using the A5-MiSeq pipeline (version 20160825). The assembled draft genome (genome size = 4,188,006 bp; 53 contigs; *N*_50_ = 308,667) was annotated by PROKKA (version 1.14.0), which predicted 4,428 putative genes. Specific primers for isolate CJ were designed using Primer-BLAST (58). The designed primer pair (CJf-CAATAGGGGATTGATCTTCCAC, CJr-ATGGACGTTAACCCGATCCCG) spans the margins and an intergenic spacer between two predicted genes (annotated as *Xre 3* and an uncharacterized gene). This primer pair was then applied for quantitative PCR.

DNA extracted from augmented and control plants was used as a template for quantitative PCR using the SYBR Green method (Fast SYBR Green; Applied Biosystems, Waltham, MA). Each reaction tube contained 3 μL DDW, 5 μL Fast SYBR Green Master Mix (Thermo Fisher Scientific), 0.5 μL of each primer (5 pmol), and 1 μL DNA. qPCR was performed for 40 cycles of 95°C for 15 sec and 64°C for 30 sec. To examine the quality and quantity of DNA, each sample was also quantified for the ELF gene of chickpeas using specific primers (ABCTf TCACAGGTTGTGATGGAGTCTG, ABCr CCTCAAATCTTGTTGGGGTGTC). SPSS was used for ANOVA statistical analysis of the qPCR results.

### Data availability.

The 16S rRNA gene sequence of isolate CJ was deposited in the NCBI database under accession number ON141592. Raw sequence data as fastq formatted files were deposited into NCBI SRA database under accession number PRJNA780499, BioSample accession numbers SAMN23247248 to SAMN23247332.
